# Association between the *CTLA4* +49A/G (rs231775) and CT60 (rs3087243) gene variants with vitiligo: study on a Mexican population^☆^

**DOI:** 10.1016/j.abd.2021.10.012

**Published:** 2022-09-24

**Authors:** Mauricio Andrés Salinas-Santander, Víctor de Jesús Suárez-Valencia, Mayela del Ángel-Martínez, David Emmanuel Kubelis-Lopez, Natalia Aranza Zapata-Salazar, Jorge Alejandro Ocampo-Garza, Jorge Ocampo-Candiani

**Affiliations:** a Research Department, Facultad de Medicina Unidad Saltillo, Universidad Autónoma de Coahuila, Saltillo, Coahuila, Mexico; b Dermatology Service, Hospital Universitario Dr José Eleuterio González, Universidad Autónoma de Nuevo León, Monterrey, Nuevo León, Mexico

**Keywords:** CTLA4, gene variants, Mexican population, Vitiligo

## Abstract

**Background:**

Vitiligo is characterized by an autoimmune response targeting melanocytes, thus resulting in skin depigmentation. There are several genetic components involved in the development of vitiligo, of which various gene polymorphisms are currently considered as risk factors. For example, the *CTLA4* (T-lymphocyte antigen 4) +49A/G (rs231775) and CT60 (rs3087243) gene variants have been associated with a predisposition for autoimmune diseases in different populations; however, their involvement in the development of vitiligo remains controversial.

**Objective:**

We evaluated the association between vitiligo and the *CTLA4* +49A/G (rs231775) and CT60 (rs3087243) gene variants in a Mexican population.

**Methods:**

A total of 116 vitiligo patients and 117 control subjects from northeast Mexico were included in the study and analyzed through PCR-RFLP to determine whether there is an association between vitiligo and *CTLA4* +49A/G (rs231775) and CT60 (rs3087243) gene variants.

**Results:**

No statistical difference was observed for both gene polymorphisms between vitiligo patients and controls (p > 0.05). Otherwise, vitiligo activity, family history of vitiligo, personal history of autoimmune diseases, or sex did not show any difference (p > 0.05).

**Conclusion:**

As suggested by the analysis of a northeastern Mexican population, the *CTLA4* +49A/G (rs231775) and CT60 (rs3087243) gene variants do not constitute a risk factor in the development of vitiligo.

## Introduction

Affecting ∼0.5%‒2% of the world population, and with a global prevalence of ∼0.06%–8.8%,^1^, ^2^ vitiligo is an autoimmune disorder characterized by the selective Oss of melanocytes that results in skin depigmentation. The development of vitiligo has a strong genetic component and several gene polymorphisms have been associated with the autoimmune response and melanogenesis typically observed in this disease.^2^ During this process, the melanocytes, the melanin cells in the epidermis, are destroyed by T-cells, which constitute one of the key mediators in the autoimmune response creating areas of depigmentation.^3^ A member of the immunoglobulin superfamily, the cytotoxic T-Lymphocyte Antigen 4 (*CTLA4*) gene, found in locus 2q33,^4^ encodes a key inhibitory receptor whose function is that of a potent negative regulator of T-cell response during the priming phase of the immune response.^5^ Therefore, *CTLA4* gene variants have been associated with several autoimmune diseases, such as autoimmune thyroid disease, Graves’ disease, Hashimoto’s thyroiditis, Type 1 diabetes,^6^ psoriasis,^7^ Behçet’s disease,^8^ and alopecia areata, although the latter remains controversial.^9^

Two *CTLA4* genetic variants have been linked with the development of vitiligo, *i.e.,* +49A/G (rs231775), an exon 1 missense variation leading to a threonine to alanine substitution at codon 17 (T17A); and CT60 A/G (rs3087243), located 236 bp downstream of the *CTLA4* 3′-UTR.^10^ However, these variants and their association with vitiligo vary widely and seem to be heavily dependent upon the analyzed population.^10^, ^11^, ^12^

Thus, this study explores whether the *CTLA4* +49 A/G and CT60 A/G gene variants are associated with vitiligo in a Mexican population.

## Material and methods

### Subjects

Patients with vitiligo and healthy control subjects were recruited from the Dermatology Department of ‘Dr. Jose E. Gonzalez’ University Hospital of the Universidad Autónoma de Nuevo León (UANL; Monterrey, Mexico). The subjects originated from the northeastern region of Mexico, including the states of Coahuila, Nuevo León, San Luis Potosí, Tamaulipas, and Zacatecas. A total of 116 vitiligo patients (62 females and 54 males; mean age of 43.06 ± 16.34 years) and 117 healthy controls (75 females and 42 males; mean age of 29.01 ± 12.67 years) were included in this study. All of the patients were directly evaluated and surveyed by a dermatologist. Vitiligo activity was determined by the time interval between the manifestation of new depigmented areas (Vitiligo with lesion stability of >1 year) or enlargement of already existing ones. This study was approved by the Ethics and Research Committee of the “Dr. Jose Eleuterio Gonzalez” University Hospital-UANL (code DE08-008). All the participants provided their informed consent.

### DNA isolation

Genomic DNA was isolated from peripheral venous blood from both vitiligo patients and control subjects. The samples were centrifuged, and the buffy-coat was processed for DNA isolation following a salting-out method, resuspending the DNA pellet in Tris-EDTA (pH 7.8) at a final concentration of 0.1–1.0 μg/μl.

### Genotyping the *CTLA4* +49A/G (rs231775) and CT60 (rs3087243) variants

The allele frequency of the *CTLA4* rs231775 and rs3087243 gene variants was characterized by PCR-RFLP using an MJ Mini PTC1148 thermal cycler (Bio-Rad, Hercules; CA, USA), the oligonucleotide primers for *CTLA4* rs231775 (5′-CCA CGG CTT CCT TTC TCG TA-3′ and 5′-AGT CTC ACT CAC CTT TGC AG-3′) and *CTLA4* rs3087243 (5′-ATG AGT CAG CTT TGC ACC AGC CAT TAC-3′ and 5′-GAG GTG AAG AAC CTG TGT TAA ACA GCA TG-3′) were obtained from IDT (IA; USA). According to a previously published protocol, the enzymes *Bbv*I and *Nla*III (New England Biolabs, MA; USA) were used in the restriction analysis.^13^ The amplicon fragments were resolved by electrophoresis in a 2.5% agarose gel, stained with ethidium bromide, and visualized in a UVP model 2UV High-Performance Transilluminator (Upland, CA, USA).

### Statistical analysis

The sample size was based on the reported incidence of vitiligo in Mexico (4%).^2^ Assuming a statistical power of 99.0% (Z = 2.33), a minimum of 84 subjects was sufficient for an accurate genetic analysis. The SPSS v21.0 software for windows (IBM, IL; USA) and the Epi-INFO™ 7 statistical program (CDC, USA) were used in the statistical analysis. A Hardy-Weinberg equilibrium test was obtained for the alleles using a goodness-of-fit test, whereas the genotypic dependence between patients and control subjects was determined with a χ *^2^* test. The OR was calculated from 2 × 2 contingency tables. A p < 0.05 was considered significant for all tests.

## Results

### Clinical parameters

The 116 vitiligo patients included in this study were classified into four categories according to the clinical presentation of the disease: 101 (87.07%) Vitiligo Vulgaris (VV), 12 (10.35%) Focal Vitiligo (FV), 2 (1.72%) Universal Vitiligo (UV), and 1 (0.86%) Segmental Vitiligo (SV) (Table 1). The age of onset, the appearance of the skin lesions after trauma (Koëbner phenomenon), and common comorbidities are shown in Table 1, Table 2. Approximately 47% of the patients had at least one relative with vitiligo, whereas the age of onset was before 30 years for 58.48% of the patients.

**Table 1:** Clinical parameters of the vitiligo patients.

Vitiligo Type	Gender, n (%)		Vitiligo Activity, n (%)		Koëbner phenomenon	Age of Onset	
	Female	Male	Active	Stable		Before 30 years, n (%)	After 30 years, n (%)
Vulgaris (VV)	56(48.28%)	45(38.79%)	45 (38.79%)	56 (48.28%)	32 (27.59%)	61 (52.59%)	40 (34.48%)
Universal (UV)	1 (0.86%)	1 (0.86%)	‒	2 (1.72%)	‒	1 (0.86%)	1 (0.86%)
Focal (FV)	4 (3.45%)	8 (6.90%)	3 (2.59%)	9 (7.76%)	2 (1.72%)	6 (5.17%)	6 (5.17%)
Segmental (SV)	1 (0.86%)	‒	1 (0.86%)	‒	‒	1 (0.86%)	‒

**Table 2:** Autoimmune diseases associated with vitiligo.

		Autoimmune diseases, n (%)			
Vitiligo Type	Thyroid	AA	T2DM	HTN	Atopy
Vulgaris (VV)	19 (16.38)	3 (2.59)	10 (8.62)	18 (15.52)	3 (2.59)
Universal (UV)	^−^	^−^	^−^	^−^	1 (0.86)
Focal (FV)	2 (1.72)	1 (0.86)	^−^	1 (0.86)	^−^
Segmental (SV)	^−^	^−^	^−^	^−^	1 (0.86)

AA, Alopecia Areata; T2DM, Diabetes Mellitus Type 2; HTN, Arterial Hypertension; VV, Vulgaris Vitiligo; UV, Universal Vitiligo; FV, Focal Vitiligo; SV, Segmental Vitiligo.

Of the vitiligo patients, 21 (18.1%) had a personal history of thyroid alterations (Table 2), of which hypothyroidism was the most prevalent (8.6%). However, type 2 diabetes mellitus (T2DM, 8.62%) and Arterial hypertension (HTN, 16.38%) were the most common comorbidities found in these patients.

### Correlation between *CTLA4* gene variants and vitiligo

We investigated whether the *CTLA4* +49A/G (rs231775) and CT60 (rs3087243) gene variants are associated with vitiligo in a sample of Mexican patients and healthy control subjects. However, no deviation was detected from the Hardy-Weinberg equilibrium for either of the evaluated *CTLA4* polymorphisms ([rs231775: Vitiligo patients Pearson 0.889, Likelihood-ratio 0.889, and Fisher exact 1; controls Pearson 0.885, Likelihood-ratio 0.885, and Fisher exact 1], [rs3087243: Vitiligo patients Pearson 0.837, Likelihood-ratio 0.837, and Fisher exact 1; controls Pearson 0.721, Likelihood-ratio 0.721, and Fisher exact 0.85]).

The comparison of *CTLA4* +49A/G (rs231775) and CT60 (rs3087243) genotypes and/or allele frequencies among the cohort of cases and control subjects, revealed that the heterozygous AG genotype (rs231775 and rs3087243) had a greater frequency across the cohort; regardless, no clear correlation between the evaluated polymorphisms and risk of developing vitiligo could be observed (p > 0.05) (Table 3). Further, no association was found between genotypes and vitiligo activity, family history, personal history of autoimmune diseases, or gender, in the analyzed patients (p > 0.05) (Table 4).

**Table 3:** Frequencies of *CTLA4* +49A/G (rs231775) and CT60 (rs3087243) alleles and genotypes in patients with vitiligo and healthy control subjects.

Genotype	Vitiligo, n (%)	Control, n (%)	χ*^2^*	OR	95% CI	p-value
All vitiligo types	rs231775					
AA	42 (36.2)	33 (28.2)	2.034			0.362
AG	55 (47.4)	59 (50.4)				
GG	19 (16.4)	25 (21.4)				
AG + GG	55 (47.4) + 19 (16.4)	59 (50.4) + 25 (21.4)	1.709	1.445	0.831‒2.511	0.191
Alleles						
A	139 (59.9)	125 (53.4)	2.001	1.303	0.902‒1.881	0.157
G	93 (40.1)	109 (46.6)	2.001	0.767	0.531‒1.108	0.157
Active Vitiligo						
AA	20 (40.8)	33 (28.2)	3.135			0.209
AG	18 (36.7)	59 (50.4)				
GG	11 (22.5)	25 (21.4)				
AG + GG	18 (36.7) + 11 (22.5)	59 (50.4) + 25 (21.4)	2.527	1.756	0.874‒3.527	0.112
Alleles						
A	58 (59.2)	125 (53.4)	0.928	1.264	0.784‒2.039	0.335
G	40 (40.8)	109 (46.6)	0.928	0.791	0.491‒1.275	0.335
Stable vitiligo						
AA	22 (32.8)	33 (28.2)	2.605			0.272
AG	37 (55.2)	59 (50.4)				
GG	8 (12.0)	25 (21.4)				
AG + GG	37 (55.2) + 8 (12.0)	59 (50.4) + 25 (21.4)	0.436	1.244	0.650‒2.382	0.509
Alleles						
A	81 (60.4)	125 (53.4)	1.708	1.333	0.866‒2.051	0.191
G	53 (39.6)	109 (46.6)	1.708	0.750	0.488‒1.155	0.191
Vulgaris vitiligo						
AA	37(36.6)	33 (28.2)	1.947			0.378
AG	47 (46.5)	59 (50.4)				
GG	17 (16.9)	25 (21.4)				
AG + GG	47 (46.5) + 17 (16.9)	59 (50.4) + 25 (21.4)	1.767	1.472	0.831‒2.605	0.184
Alleles						
A	121 (59.9)	125 (53.4)	1.853	1.303	0.890‒1.907	0.173
G	81 (40.1)	109 (46.6)	1.853	0.768	0.525‒1.124	0.173
All vitiligo types	rs3087243					
AA	26 (22.4)	20 (17.1)	1.488			0.475
AG	59 (50.9)	59 (50.4)				
GG	31(26.7)	38 (32.5)				
AG + GG	59 (50.9) + 31 (26.7)	59 (50.4) + 38 (32.5)	1.040	1.401	0.732‒2.683	0.308
Alleles						
A	111 (47.8)	99 (42.3)	1.443	1.251	0.868‒1.803	0.230
G	121 (52.2)	135 (57.7)	1.443	0.799	0.555‒1.152	0.230
Active vitiligo						
AA	13 (26.5)	20 (17.1)	1.995			0.369
AG	21 (42.9)	59 (50.4)				
GG	15 (30.6)	38 (32.5)				
AG + GG	21 (42.9) + 15 (30.6)	59 (50.4) + 38 (32.5)	1.931	1.751	0.790‒3.883	0.165
Alleles						
A	47 (48.0)	99 (42.3)	0.895	1.257	0.783‒2.018	0.344
G	51 (52.0)	135 (57.7)	0.895	0.796	0.496‒1.278	0.344
Stable vitiligo						
AA	13 (19.4)	20 (17.1)	1.519			0.469
AG	38 (56.7)	59 (50.4)				
GG	16 (23.9)	38 (32.5)				
AG + GG	38 (56.7) + 16 (23.9)	59 (50.4) + 38 (32.5)	0.154	1.168	0.539‒2.531	0.694
Alleles						
A	64 (47.8)	99 (42.3)	1.027	1.247	0.814‒1.911	0.311
G	70 (52.2)	135 (57.7)	1.027	0.802	0.523‒1.229	0.311
Vulgaris vitiligo						
AA	21 (20.8)	20 (17.1)	1.039			0.595
AG	53 (52.5)	59 (50.4)				
GG	27 (26.7)	38 (32.5)				
AG + GG	53 (52.5) + 27 (26.7)	59 (50.4) + 38 (32.5)	0.486	1.273	0.645‒2.513	0.486
Alleles						
A	95 (47.0)	99 (42.3)	0.979	1.211	0.829‒1.769	0.322
G	107 (53.0)	135 (57.7)	0.979	0.826	0.565‒1.207	0.322

**Table 4:** Correlation between *CTLA4* +49A/G (rs231775) and CT60 (rs3087243) gene polymorphisms with vitiligo activity, family history, personal history of autoimmunity, and gender.

		+49A/G (rs231775)				CT60 (rs3087243)			
		AA	AG	GG	p-value	AA	AG	GG	p-value
Vitiligo activity	Active	20	18	11	0.108	13	21	15	0.335
	Stable	22	37	8		13	38	16	
Family history of vitiligo	Yes	20	24	11	0.562	12	26	17	0.617
	No	22	31	8		14	33	14	
Personal history of autoimmune diseases	Yes	17	19	11	0.202	12	23	12	0.802
	No	25	36	8		14	36	19	
Sex	Female	20	31	11	0.634	12	33	17	0.695
	Male	22	24	8		14	26	14	

## Discussion

Vitiligo is a complex skin disorder in which the melanocytes are progressively and selectively destroyed.^14^ Multiple internal and external factors have been associated with the development of vitiligo, including autoimmune alterations, genetic factors, epidermal trauma, emotional stress, infections, among other risk factors that could act independently of each other or in combination.^2^, ^15^ In addition, it is thought that vitiligo may have a non-Mendelian hereditary pattern, incomplete penetrance, multiple susceptibility loci, and genetic heterogeneity.^16^

Vitiligo has been associated with multiple gene variants, several of which are involved in immune response pathways, mainly in relation with the Human Leukocyte Antigen (HLA) class I and class II gene regions of the Major Histocompatibility Complex (MHC) and non-MHC candidate genes.^17^ Regarding the latter, the *CTLA4* gene encodes a T-cell co-receptor expressed by both CD4+ and CD8+ T-cells, which is involved in T-cell activation; further, this co-receptor is a critical negative regulator of T-cell response, thus playing an essential protection role against autoimmunity.^18^ The autoimmune response mediated by CD8+ T-cells is key during the depigmentation process of vitiligo, as these cells are directly responsible for the destruction of the melanocytes, creating the typically observed areas of skin depigmentation.^3^

The association between the *CTLA4* +49 G/A and CT60 gene variants and the development of different autoimmune diseases have been previously suggested. 19, 20 However, the results have been inconsistent in dermatological diseases; for instance, in cases of psoriasis, their participation or lack of influence has been reported.^21^ A similar condition has been observed in Alopecia Areata (AA), where a previous association study proposed the involvement of *CTLA4* polymorphisms as a risk factor in its development in a European population;^22^ however, another study revealed that these polymorphisms were irrelevant for the development of AA in a Mexican population.^9^

In Latin America, *CTLA4* genetic variants have only been correlated with the development of obesity in northeastern Brazil,^23^ type 1 diabetes mellitus in Chile,^24^ FVIII inhibitor development in hemophilia A (HA) patients from Argentina,^25^ diffuse cutaneous leishmaniasis in Venezuela, ^26^ and hepatitis C virus^27^ and rheumatoid arthritis in Mexico.^28^

Although the participation, or lack of influence, of both gene polymorphisms has been reported for vitiligo, a meta-analysis study including 1252 cases in four European, three Asian, and two Turkish populations, revealed that the *CTLA4* CT60 A/G gene variants confers susceptibility to vitiligo only in the European population.^10^

In previous studies, we observed the role of TNFα ^29^ and PTPN22 https://www.sciencedirect.com/science/article/pii/S0365059620300921 - bib0195 ^30^ genetic variants as risk factors in the development of active forms of vitiligo in a Mexican population. Both genes are related to the regulation of immune mechanisms. However, the influence of *CTLA4* gene variants in the development of vitiligo has not been analyzed in Mexican patients. Perhaps unsurprisingly, this study has shown no association of either rs231775 or rs3087243 polymorphisms with vitiligo, suggesting that these gene variants do not constitute a major risk factor in this regard within a northeastern Mexican population.

## Conclusion

In conclusion, the *CTLA4* gene variants rs231775 and rs3087243 do not constitute a risk factor in the development of vitiligo in the analyzed northeastern Mexican population. Further, these genetic variants do not correlate with the personal history of autoimmune diseases, family history of vitiligo, or the sex of the included subjects.

## Financial support

This research did not receive any specific grant from funding agencies in the public, commercial, or not-for-profit sectors.

## Authors’ contributions

Mauricio Salinas-Santander: Study conception, design, and planning; collection, analysis, and data interpretation; writing; critical literature review and critical review of the manuscript; approval of the final version of the manuscript.

Víctor Suárez-Valencia: Analysis, and data interpretation; writing; critical literature review and critical review of the manuscript; approval of the final version of the manuscript.

Mayela del Ángel-Martínez: Data interpretation; writing; critical literature review and critical review of the manuscript; approval of the final version of the manuscript.

David Kubelis-Lopez: Data interpretation; critical review of the manuscript; approval of the final version of the manuscript.

Natalia Zapata-Salazar: Data interpretation; critical review of the manuscript; approval of the final version of the manuscript.

Jorge Ocampo-Garza: Critical review of the manuscript; approval of the final version of the manuscript.

Jorge Ocampo-Candiani: Critical review of the manuscript; approval of the final version of the manuscript.

## Conflicts of interest

None declared.
